# *SRY* and *NR5A1* gene mutation in Algerian children and adolescents with DSD and testicular dysgenesis

**DOI:** 10.4314/ahs.v21i3.61

**Published:** 2021-09

**Authors:** Naouel Kherouatou-Chaoui, Djalila Chellat-Rezgoune, Mohamed Larbi Rezgoune, Ken Mc Elreavey, Laaldja Souhem Touabti, Noreddine Abadi, Dalila Satta

**Affiliations:** 1 Laboratory of Cellular and Molecular Biology. Frères Mentouri University-Constantine 1, Constantine, Algeria; 2 Human Genetic Developmental Unit, Pasteur Institute, Paris, France; 3 Pediatric Urology Unit, C.H.U Sétif, Sétif, Algeria; 4 Laboratory of Biology and Molecular Genetic, University Constantine 3, Constantine, Algeria

**Keywords:** DSD, consanguinity, karyotyping, SRY, NR5A1, sequencing

## Abstract

**Background:**

In humans, sex determination and differentiation is genetically controlled. Disorders of sex development (DSD) result in anomalies of the development of the external and internal genitalia. Variants in transcription factors such as SRY, NR5A1 and SOX9, can cause changes in gonadal development often associated with ambiguity of the external genitalia.

**Objectives:**

This study has been conducted to determine the frequency, types and associated genetic alterations in patients with DSD in the Algerian population.

**Methods:**

Thirty patients were included. Based on their clinical presentation, thirteen patients presented with ambiguous external genitalia, thirteen patients presented with hypospadias and four patients presented with bilateral undescended testes. Karyotype analysis was performed on peripheral blood lymphocytes using standard R-banding. DNA was isolated from blood leukocytes for PCR reaction and mutational analysis of SRY and NR5A1 was done by direct sequencing.

**Results:**

Most patients with ambiguous genitalia had a 46,XY karyotype. One patient had a deletion of SRY, otherwise no point mutations in SRY or NR5A1 genes were identified. However, a single NR5A1 polymorphism (p.Gly146Ala) in patient with 46,XX DSD has been detected.

**Conclusions:**

The absence of mutations in these genes suggests that there are others genes playing an important role in sex development and differentiation.

## Introduction

Disorders of sex development DSD comprise a series of genetic diseases in which chromosomal, gonadal, or anatomical sex is atypical^1^. Ambiguous genitalia are present in about 1 in 2,000 live newborns and reflects a situation where the external genitalia cannot be established as being either male or female. Four steps must occur during sexual development and differentiation: establishment of chromosomal sex at fertilization, formation of undifferentiated gonads, gonadal differentiation into testes or ovaries, and development of the internal and external genitalia^2^.

Error in any of these stages may lead to DSD, which can be classified into the following groups: 46,XX DSD (overvirilisation or masculinisation of a 46,XX female), 46,XY DSD (undervirilisation or undermasculinisation of an XY male), Ovotesticular DSD, 46,XX Testicular DSD (XX male), and 46,XY Complete Gonadal Dysgenesis (CGD)^1^,^3^.

Mutations in several genes are known to cause DSD. Most often the underlying cause of DSD is a variant in a gene or genes regulating gonadal/genital or steroidogenic pathways. The commonest known genetic condition that leads to 46,XX DSD is congenital adrenal hyperplasia (CAH) due to 21α-hydroxylase deficiency. However Among the 46,XY DSD, the most common cause is androgen insensitivity syndrome (AIS) with mutations in the androgen receptor gene^1^.

The Sox (SRY-type HMG box) genes encode a group of proteins characterized by the presence of an HMG-box, a 79 amino acid motif which can bind and bend DNA, which is the only part of the testis-determining gene SRY that is conserved between species^4^. Mutations in SRY are associated with 46,XY complete gonadal dysgenesis and are found in approximately 15% of these cases^5^. Most of the mutations detected are located in the HMG domain of the protein. The second most common monogenic cause of 46,XY DSD are mutations involving the NR5A1 gene. NR5A1 regulates multiple genes involved in adrenal and gonadal development steroidogenesis, reproduction and other metabolic functions^6^,^7^. Mutations in the human NR5A1 gene has a phenotypic spectrum that ranges from complete testicular dysgenesis with Müllerian structures, through individuals with mild clitoromegaly or genital ambiguity, to severe penoscrotal hypospadias or even anorchia^8^–^14^. Although not essential for ovarian determination^15^, heterozygote mutations in NR5A1 have recently been linked to ovarian insufficiency, thus confirming the role of this factor in ovarian follicologenesis 16,17. A link between NR5A1 mutations and the occurrence of male infertility was also found^18^.

Recently, a recurrent NR5A1 p.Arg92Trp mutation was also identified in several patients with 46,XX testicular/ovotesticular DSD, highlighting the role of NR5A1 in the development of both testes and ovaries^19^.

In view of the importance of the SRY and NR5A1 genes in normal gonadal development, this first study in Algeria was undertaken with an aim to screen for mutations in these genes in DSD Algerian cases and to describe their clinical and cytogenetics featuresing.

## Materials and Methods

### Patients

Informed consent was obtained from all subjects participating in the study. Thirteen patients with ambiguous external genitalia, thirteen patients with hypospadias and four patients with bilateral testicular ectopy were included. Twenty-eight were children aged 22 days-13 years and two were young adult aged 18-20 years. All patients were of Algerian origin and from different geographic locations. These patients came to our laboratory for karyotype analyses. The controls were normal adult males with 46,XY and females with 46,XX chosen from volunteers. A blood sample was obtained from each subject for karyotype analysis, hormonal analysis and DNA extraction.

### Cytogenetic analysis

Peripheral blood lymphocyte cultures were set up using TC199 medium for human lymphocyte culture for 72 hours. Dividing cells were arrested at metaphase stage with Colcemide and fixed using Carnoy acetic solution after treatment with hypotonic solution according to standard procedures. The harvested cells were dropped on clean slides and the chromosomes were studied by RHG method (R-bands after heat denaturation and Giemsa). A total of 30 metaphases were analyzed for each sample and the karyotypic descriptions were according to the ISCN recommendations to look at any numerical or structural chromosomal aberrations 20.

### PCR and sequencing the coding region of candidate genes

Previously we have analyzed the presence of the SRY gene in leucocytes in all cases by PCR. The complete open reading frames of SRY and each exon of NR5A1 were amplified by polymerase-chain-reaction (PCR) according to a previous report^16^,^21^. After amplification, PCR products were electrophoresed in a 2% agarose gel and afterwards the PCR products were purified using exonuclease I and phosphatase alkaline. These products were then sequenced (10-15ng DNA template reaction) on an Automated DNA sequencer using the big dye V1.1 terminator cycle sequencing. Cycling conditions were as follows: initial denaturation at 96°C for 1min and 25 cycles at 96°C for 10 sec, 50°C for 5 s, and 60 °C for 4 min. DNA sequencing of both sense and antisense strands were carried out for all exons of the candidate genes.

### Ethics

Ethics approval was obtained from the Ethics Committee of the university hospital center Benbadis of Constantine.

## Results

### Karyotype and Molecular Analysis

Table 1 shows the karyotype in the 30 cases with sex anomalies. Thirteen of these patients (43.33%) were referred as cases with ambiguous genitalia. Nine of them (30%) had 46,XY constitution, among these cases three had uterus. Three of them had 46,XX constitution (10%), one had uterus and ovaries/penis and testicles. The remaining case (3%) with ambiguous genitalia had peripheral blood karyotype of 47,XYY, ectopic testicles, penis and labia Majora. Thirteen cases (43.33%) had been referred as patients with hypospadias with or without undescended testes with 46,XY constitution. The four remaining cases (13.33%) with undescended testes only had peripheral blood karyotype of 46,XY.

**Table 1:** Summary of all characteristics of included patients (n=30)

Patient	Age	Phenotype	Clinical data	Karyotype	SRY gene	NR5A1 gene	Consanguinity	Endocrine data
1	13 years	**Male Partial gonadal** **dysgenesis**	Hypoplastic Uterus	46,XY	Positive	Normal	+	N
2	8 years	**Female Partial** **gonadal dysgenesis**	ambiguous external genitalia with bilateral inguinal hernia Uterus / ovaries	46,XY	Negative	Normal		T(N), FSH↑, LH↑
3	22 days	**Male Partial gonadal** **dysgenesis**	Micropenis Enlarged labia majora Hypoplastic uterus	46,XY	Positive	Normal		T↓ at birth
4	5 years	**Ambiguous genitalia** **raised as a boy**	Ambiguity of external genitalia Micopenis	46,XY	Positive	Normal		T ↓, FSH (N), LH ↓
5	20 years	**Ambiguous genitalia** **raised as a boy**	Prostate Ectopic testicles/ Penis Labia majora	47,XYY	Positive	Normal		T(N), FSH ↑, LH↑
6	18 years	**Ambiguous genitalia** **raised as a boy**	Ambiguity of external genitalia	46,XY	Positive	Normal		T(N), FSH ↑, LH↑
7	10 years	**Ambiguous genitalia** **raised as a boy**	Ambiguity of external genitalia	46,XY	Positive	Normal		T ↓, FSH (N), LH ↓
8	4 years	**Ambiguous genitalia** **raised as a boy**	Left testicle absent Right ectopic testicle Micropenis	46,XY	Positive	Normal		T ↓, FSH (N), LH ↓
9	3 months	**Ambiguous genitalia** **raised as a boy**	Ambiguity of external genitalia	46,XY	Positive	Normal		T(N), FSH (N), LH ↓
10	23 days	**Ambiguous genitalia** **raised as a boy**	Ambiguity of external genitalia	46,XY	Positive	Normal		NA
11	4 years	**Male** **with Hypospadias**	Posterior hypospadias	46,XY	Positive	Normal		N
12	13 months	**Male** **with Hypospadias**	Vulviform hypospadias	46,XY	Positive	Normal		N
13	5 years	**Male** **with Hypospadias**	Posterior hypospadias	46,XY	Positive	Normal		N
14	4 years	**Male** **with Hypospadias**	Vulviform hypospadias Renal malformation	46,XY	Positive	Normal	+	N
15	3 years	**Male** **with Hypospadias**	Posterior hypospadias	46,XY	Positive	Normal		N
16	4 years	**Male** **with Hypospadias**	Posterior hypospadias	46,XY	Positive	Normal	+	N
17	5 years	**Male** **with Hypospadias**	Posterior hypospadias	46,XY	Positive	Normal	+	N
18	5 years	**Male** **with Undescended** **testis**	Bilateral undescended testes	46,XY	Positive	Normal		N
19	5 years	**Male** **with Undescended** **testis**	Bilateral undescended testes	46,XY	Positive	Normal		N
20	2 years	**Male** **with Undescended** **testis**	Bilateral undescended testes	46,XY	Positive	Normal		N
21	1 year	**Male** **with Undescended** **testis**	Bilateral undescended testes	46,XY	Positive	Normal		N
22	15 months	**Male** **with Hypospadias and** **undescended testis**	Posterior hypospadias with Bilateral undescended testes	46,XY	Positive	Normal	+	N
23	4 years	**Male** **with Hypospadias and** **undescended testis**	Posterior hypospadias with Bilateral undescended testes Micropenis	46,XY	Positive	Normal		N
24	3 years	**Male** **with Hypospadias and** **undescended testis**	Vulviform hypospadias with Chordee Bilateral undescended testes	46,XY	Positive	Normal		N
25	3 years	**Male** **with Hypospadias and** **undescended testis**	Vulviform hypospadias with Bilateral undescended testes	46,XY	Positive	Normal		N
26	2 years	**Male** **with Hypospadias and** **undescended testis**	Posterior hypospadias with Bilateral undescended testes	46,XY	Positive	Normal		N
27	5 years	**Male** **with Hypospadias and** **undescended testis**	Vulviform hypospadias with Bilateral undescended testes	46,XY	Positive	Normal		N
28	13 years	**Male ovotesticular** **DSD**	Uterus Ovaries/Penis /Testicles Hypospadias	46,XX	Negative	Normal	+	T ↑,FSH (N), LH ↓
29	13 years	**46,XX virilised (gonad** **status unknown)**	Ambiguity of external genitalia	46,XX	Negative	c.437G→C) polymorphism		T ↑,FSH↓, LH↓
30	6 years	**46,XX virilised (gonad** **status unknown)**	Ambiguity of external genitalia	46,XX	Negative	Normal		T ↓,FSH↑, LH ↑

Age- and sex-dependent hormone levels in the normal population are taken as given previously 22.

Table 1 showed that six cases (20%) of the 30 had positive consanguinity. Cases 16 and 17 were brothers. The presence or absence of SRY gene for all cases was reported above in Table 1. One case of 46,XY gonadal dysgenesis carried a deletion of SRY. This patient has ambiguous external genitalia with bilateral inguinal hernia and female's internal reproductive organs (uterus and ovaries).

### *SRY* and *NR5A1* Genes Direct Sequencing

The SRY gene had only one exon. One pair of primer was designed for this exon. The whole coding sequence of SRY gene was sequenced but no mutation or deletion was found in all patients whose SRY gene was positive.

In the 30 cases with DSD we did not detect a mutation in the whole coding sequence of NR5A1 gene. We identified a single NR5A1 polymorphism. This patient presented with 46,XX DSD. The polymorphism consisted of a G to C transversion at nucleotide position 437 in exon 3 that is predicted to result in a p.Gly146Ala amino acid change.

## Discussion

DSD ranging from minor genital abnormalities to complete sex-reversal are the most common birth defects with an incidence rate of almost 3%^23^. Genetic studies of human patients presenting with DSD have revealed an increasing number of genes important of sex determination and differentiation. Our genetic study was divided into two main categories: karyotyping and molecular study. Karyotyping of the 30 patients revealed a normal female karyotype 46,XX for 3 cases and a normal male karyotype 46,XY for 26 cases and one karyotype with anomalies in the sex chromosomes 47,XYY. This is in contradiction with the finding of Abou El Ella et al.^24^ and Anhalt et al.^25^, who reported that the most common presentation of DSD was females (77% and 70% of cases, respectively). Karyotyping is essential for provisional diagnosis and classification of the DSD cases^26^. Consanguinity was found in 6 cases of our 30 cases (20%). Two cases with hypospadias and ectopic testis were brothers. It is important to note that the most of the published data on the incidence of DSD available from Western countries are with low rates of consanguinity, and it may not be a true reflection of the worldwide prevalence. There is, however, some evidence for a higher rate of DSD in societies with a higher rate of consanguinity^27^. In this context, Abou El Ella et al.^24^ and Shawky et al.^28^ found a consanguinity rate of 61.54% and 35.3%, respectively. A retrospective study in Sudan, of the 122 cases of DSD, found that 69 cases were 46,XX DSD and 45 cases were 46,XY DSD. The most common cause of 46,XX DSD was CAH 21-hydroxylase deficiency, whereas androgen insensitivity was the most frequent cause of DSD in 46,XY individuals. 70% of all 122 cases were born to first cousin marriages^29^. A study of 26 cases of DSD from Sudan indicated that parental consanguinity was observed in 70% of cases^30^. Consanguinity has been proposed as the cause of a high frequency of 46,XX DSD (CAH, 65.4% of total DSD cases) in one referral center in Saudi Arabia over a 20 year period^31^. In our study most of the cases of ambiguous genitalia had a 46,XY karyotype and only three cases were 46,XX.

These conflicting results indicate that consanguinity may lead to an increase in the incidence rates of both 46,XY and 46,XX DSD, depending on the population studied.

The main gene implicated in sex determination in mammals is the sex-determining region on the Y chromosome (SRY), which encodes a transcription factor that is a member of the high mobility group (HMG)-box family of DNA binding proteins and in mammals triggers the development of undifferentiated gonads towards a testicular phenotype^32^. In humans, zygotes bearing mutations in SRY develop into XY females^33^, whilst XX individuals with the presence of SRY typically show a normal male phenotype^34^ but may occasionally show ambiguous genitalia^35^. Only one of our 26 patients with 46,XY karyotype was SRY-negative. Gonadal dysgenesis in this patient was caused by the absence of the SRY gene. Two of the XX individuals were SRY-negative, but show a male phenotype and ambiguous genitalia. Most SRY-negative XX males have a high incidence of genital ambiguity or hypospadias^36^. Direct sequencing of the whole SRY coding region revealed no mutation in all patients studied. Mutations in the SRY gene have been described in patients with DSD^5^,^37^–^43^. Mutations involving the SRY gene are almost exclusively associated with complete rather than partial gonadal dysgenesis and this may explain the lack of SRY mutations in this cohort. Furthermore, in male sexual development there are many other genes, which also play a crucial role. NR5A1 is a key regulator of adrenal and reproductive development and function. Approximately, 191 variants NR5A1 have been identified in humans to date^44^. In the 30 studied cases with DSD, no mutations were detected in the NR5A1 gene. One subject who had 46, XX DSD was heterozygous for the p.Gly146Ala (c.437G→C) polymorphism that has previously been demonstrated to have approximately 80% of the activity of the wildtype protein^45^. The contribution of this variant to the development of DSD is unknown. It have been reported that this variant, was found to be very frequent in Egyptian cohort with 46, XY DSD (34 %)^46^ which is not the case in our cohort.

The limitation of the current study is small sample size and the fact that only two genes involved in the sex determination were screened.

## Conclusion

We found in Algeria that the most common form of DSD associated with ambiguous external genitalia in the newborn is 46,XY DSD. The pathology of the majority of these cases is not explained by mutations in the two most common genes associated with DSD namely, SRY and NR5A1 suggesting that other genes are involved. Finally, we anticipate in the future sequencing a panel of genes and whole exome for genetic diagnosis of Algerian patients with DSD.
